# Chloroplast Genome Assembly and Genetic Structure Analysis of *Coffea arabica* L. Based on Maternal Inheritance Markers

**DOI:** 10.3390/genes17101304

**Published:** 2026-10-20

**Authors:** Yixian Wu, Xiaoyun Gu, Yaqi Li, Dexin Liu, Xiaofei Bi, Faguang Hu

**Affiliations:** 1 Tropical and Subtropical Cash Crops Institute, Yunnan Academy of Agriculture Science, Baoshan 678000, China; 13888889644@163.com (Y.W.); 18213074851@163.com (X.G.); zzjgray@yaas.org.cn (Y.L.); susie_liu1995@126.com (D.L.); 2 School of Public Service and Management, Yunnan Communications Vocational College of Transportation, Anning 650300, China; 3 Yunnan Key Laboratory of Coffee, Baoshan 678000, China; 4 Key Laboratory of Coffee Quality and Safety, State Administration for Market Regulation, Baoshan 678000, China

**Keywords:** *Coffea arabica*, chloroplast genome, population structure, maternal lineage, haplotype, simple sequence repeat, comparative genomics

## Abstract

**Background:** Codon usage bias has been analyzed in the *Coffea arabica* L. nuclear genome, yet comprehensive genomic resources for this species remain limited. The chloroplast genome, characterized by its maternal inheritance and high conservation, represents a valuable resource for investigating plant phylogeny, crop domestication, and maternal lineage evolution. **Results:** We assembled complete chloroplast genomes for 52 *C. arabica* accessions, encompassing four cultivars (Geisha, Catimor, Bourbon, and Caturra), four improved breeding lines within the Geisha group (Yunka No. 3, Yunka No. 4, G02, and G13), two Catimor breeding lines (CCCA25 and T5175), and four accessions of unknown cultivar origin. Comparative genomics, unrooted phylogenetic reconstruction (neighbor-joining and maximum likelihood), haplotype inference, and exploratory principal component analysis (PCA) were integrated to characterize the genetic architecture of these chloroplast genomes. These genomes ranged from 155,133 bp to 155,192 bp in length, displayed the typical quadripartite architecture, and exhibited GC contents of 37.43–37.44%. Gene annotation identified 131 genes, comprising 86 protein-coding genes, 37 tRNAs, and 8 rRNAs. Codon usage analysis revealed a pronounced bias toward A or U at the third codon position. We detected 194 SSR loci per plastome, among which mononucleotide A/T repeats predominated. Neighbor-joining and maximum-likelihood phylogenies, PCA, and haplotype network reconstruction consistently resolved the 52 accessions into three genetic clusters (C1–C3; C1 represented by a single divergent accession) and five distinct haplotypes. SNP and InDel profiling further showed that intergenic spacer (IGS) regions harbored the highest density of genetic variation. **Conclusions:** These genomes provide resources for germplasm characterization, phylogenetic studies, and marker-assisted breeding in coffee. They also establish a chloroplast-genome framework that, when integrated with nuclear genomics and expanded sampling, will help fully resolve the maternal history, domestication, and allopolyploid origin of *Coffea arabica*.

## 1. Introduction

Coffee is among the most economically important beverage crops globally. A member of the Rubiaceae family, commercial cultivation centers on two species: *C. arabica* (Arabica) and *Coffea canephora* (Robusta). In China, coffee production is anchored in Yunnan Province, where the crop has emerged as a vital component of the region’s distinctive agricultural economy. Coffee quality, yield, and stress tolerance are intimately linked to its genetic constitution. Consequently, accurate germplasm identification and comprehensive assessment of genetic diversity underpin cultivar improvement and sustainable industry development.

The chloroplast genome exhibits maternal inheritance and a highly conserved quadripartite structure, with a moderate genome size and evolutionary rate suitable for phylogenetic and population genetic inference [1,2]. The typical chloroplast genome is a circular double-stranded DNA molecule comprising a large single-copy region (LSC), a small single-copy region (SSC), and a pair of inverted repeats (IRs). Compared with nuclear genomes, chloroplast markers—including haplotypes, simple sequence repeats (SSRs), and single nucleotide polymorphisms (SNPs)—effectively elucidate population history, dispersal routes, and genetic structure in plants [3,4]. Because the plastome is a single, haploid, largely non-recombining, maternally inherited molecule, chloroplast data capture maternal history only and cannot, by themselves, reveal paternal ancestry, biparental gene flow, or cytonuclear discordance.

To date, coffee research has largely focused on cultivation management, quality chemistry, and genetic analysis based on a limited number of nuclear gene markers. Large-scale population genetic studies utilizing complete chloroplast genomes remain scarce. Although a few coffee chloroplast genomes have been reported, these studies have largely relied on genome skimming of one or a few accessions coupled with basic annotation, leaving intraspecific variation patterns and population-level phylogenetic relationships largely unexplored. Here, we report high-throughput sequencing, assembly, and annotation of chloroplast genomes from 52 representative *C. arabica* accessions. We systematically analyzed genomic structural features, codon usage bias, and repeat sequence distribution, and conducted population genetic structure analysis (unrooted neighbor-joining and maximum-likelihood trees and principal component analysis), haplotype network construction, and SNP/InDel analysis. We aimed to (1) elucidate the fundamental characteristics and variation spectrum of the coffee chloroplast genome, (2) reveal the population genetic structure and differentiation of coffee germplasm, and (3) identify candidate molecular markers applicable to germplasm authentication and genetic research. These findings provide a genomic framework for evaluation, conservation, and utilization of coffee genetic resources.

## 2. Materials and Methods

### 2.1. Plant Materials and DNA Extraction

A total of 52 *C. arabica* leaf samples were collected from major coffee-producing regions in Yunnan Province, China (Baoshan, Pu’er, Lincang, and Nujiang; Table S1). These included 27 Geisha accessions (four improved breeding lines: Yunka No. 3, Yunka No. 4, G02, and G13), 12 Catimor accessions (including CCCA25 and T5175), 4 Bourbon accessions, 5 Caturra accessions, and 4 accessions of unknown cultivar origin (H_4, H_5, H_7, and H_11). In this study, ‘cultivar’ denotes a named cultivated variety, ‘accession’ an individual collected sample, and “breeding line” or ‘improved breeding line’ a named selection within a cultivar group. Several samples represent multiple plants of the same clonally maintained cultivar or breeding line and may share identical maternal haplotypes; sample-level and unique-accession-level summaries are provided in Tables S2–S4. Fresh leaves were frozen in liquid nitrogen, and genomic DNA was extracted using a modified CTAB protocol [5]. DNA integrity was assessed by 0.8% agarose gel electrophoresis using a Bio-Rad Mini-Sub Cell GT system (Bio-Rad Laboratories, Hercules, CA, USA). DNA concentration and purity were measured using a Thermo Scientific GENESYS 50 UV-Vis spectrophotometer (Thermo Fisher Scientific, Waltham, MA, USA) at 260 nm and 280 nm.

### 2.2. Chloroplast Genome Sequencing, Assembly, and Annotation

Paired-end libraries (insert size ~350 bp) were sequenced on the Illumina NovaSeq 6000 platform (150 bp paired-end reads). Raw reads were cleaned with fastp v0.20.0 [6] to remove low-quality bases and adapters. Clean reads were assembled de novo using SPAdes v3.14.1 [7] with k-mer sizes of 21, 45, 65, 85, and 105. Assembly graphs were visualized in Bandage v0.8.1 [8], and chloroplast-derived contigs were identified by BLASTN against the *C. arabica* reference chloroplast genome (NC_008535), manually ordered, and joined into circular genomes. Mean sequencing depth, recalculated by mapping all clean reads to the final assemblies, ranged from 777.4× to 5380.8× (mean ± SD = 2652.3 ± 1487.7×; Table S5). A complete assembly was defined as a full-length circular assembly containing the complete 131-gene set, all four single-copy/inverted-repeat junctions (LSC/IRB, IRB/SSC, SSC/IRA, and IRA/LSC), and no unresolved gaps or ambiguous bases (N bases = 0; Table S6). The four junctions were validated by PCR amplification and Sanger sequencing (Table S7). Annotation was performed with PGA [9] using the published *C. arabica* chloroplast genome as reference, followed by manual curation in Geneious Prime v2023.0.4; circular maps were generated with OGDRAW v1.3.1 [10]. Raw reads are available in SRA under BioProjects PRJNA1157617 and PRJNA1502542. The 52 assembled chloroplast genomes and annotations were submitted to NCBI GenBank under submission ID SUB16536206; accession numbers will be provided in Table S5 upon release. The 52 × 15 accession-by-variant matrix is provided in Table S8.

### 2.3. Genome Characterization and Repeat Analysis

The boundaries of the large single-copy (LSC), small single-copy (SSC), and two inverted repeat (IR) regions were determined based on the annotation coordinates. Genome size, overall GC content, and gene counts were calculated using a custom Perl script. Relative synonymous codon usage (RSCU) values were computed for all protein-coding sequences (CDSs) using CodonW v1.4.2. Simple sequence repeats (SSRs) were identified using MISA v1.0 [11] with the following criteria: mononucleotide repeats ≥10, dinucleotide repeats ≥5, and tri- to hexanucleotide repeats ≥3. For SSRs, the number of loci per plastome, the number of loci in each repeat class (mono- to hexanucleotide), the number of occurrences, and the number of unique motif classes across the dataset were reported separately, together with per-accession ranges and means. Dispersed repeats (including forward, reverse, complement, and palindromic repeats) were detected using REPuter [12] with a minimum repeat size of 30 bp and a Hamming distance of 3. Tandem repeats were predicted using Tandem Repeats Finder v4.09 [13] with default parameters.

### 2.4. Multiple Sequence Alignment and Variant Matrix Construction

The 52 complete chloroplast genome sequences were aligned using MAFFT v7.427 [14] with the L-INS-i strategy. The resulting multiple sequence alignment served as the basis for the following analysis. A complete 52-accession × 15-variant genotype/haplotype matrix, including genomic coordinate, reference/alternative state, genomic region, and gene/intergenic annotation, was compiled and is provided as Table S8.

### 2.5. Phylogenetic Reconstruction

A neighbor-joining (NJ) tree was constructed in MEGA7 [15] using the Kimura 2-parameter model, with bootstrap support estimated from 1000 replicates. A maximum-likelihood (ML) tree was also inferred using IQ-TREE v2.1.3 [16]; the best-fit substitution model was selected by ModelFinder [17], and branch support was assessed with 1000 ultrafast bootstrap replicates [18]. In the Results, the ML topology, the selected substitution model, and ultrafast bootstrap support values are reported and compared with the NJ tree. Because no accession of a related Coffea species was included as an outgroup, both trees are presented as unrooted.

### 2.6. Principal Component Analysis (PCA)

Genetic relationships among accessions were explored by PCA based on the genomic relationship matrix, implemented in GCTA v1.94.1 [19] Because the chloroplast genome is haploid and its variants are physically linked across a largely non-recombining molecule, SNP genotypes were encoded as 0/1 (reference/alternative) and no linkage-disequilibrium pruning was applied. All 52 accessions were analyzed and plotted individually at the accession level (not as cluster centroids), and the proportion of variance explained by PC1, PC2, and PC3 was reported on the corresponding axes. Given the physical linkage of plastome variants, PCA was used as an exploratory ordination of accession-level relationships rather than as independent evidence of population structure.

### 2.7. Haplotype Analysis

Haplotypes were inferred with DnaSP v6.12.03 [20], and a TCS haplotype network [21] was constructed in PopART v1.7 [22]. Because the TCS network is unrooted, it was used only to display mutational relationships among haplotypes, not to infer the directionality of haplotype derivation.

### 2.8. Variant Detection

Single nucleotide polymorphisms (SNPs) and insertions/deletions (InDels) were identified against the reference chloroplast genome (NC_008535) using DnaSP v6.12.03 [22] and classified as coding sequences (CDSs), introns, or intergenic spacers (IGSs). For each CDS SNV, the nucleotide change, codon change, synonymous/nonsynonymous status, and amino-acid substitution were determined from the annotated reading frame (Table S9).

## 3. Results

### 3.1. Basic Features of the Chloroplast Genome

**Figure 1:**
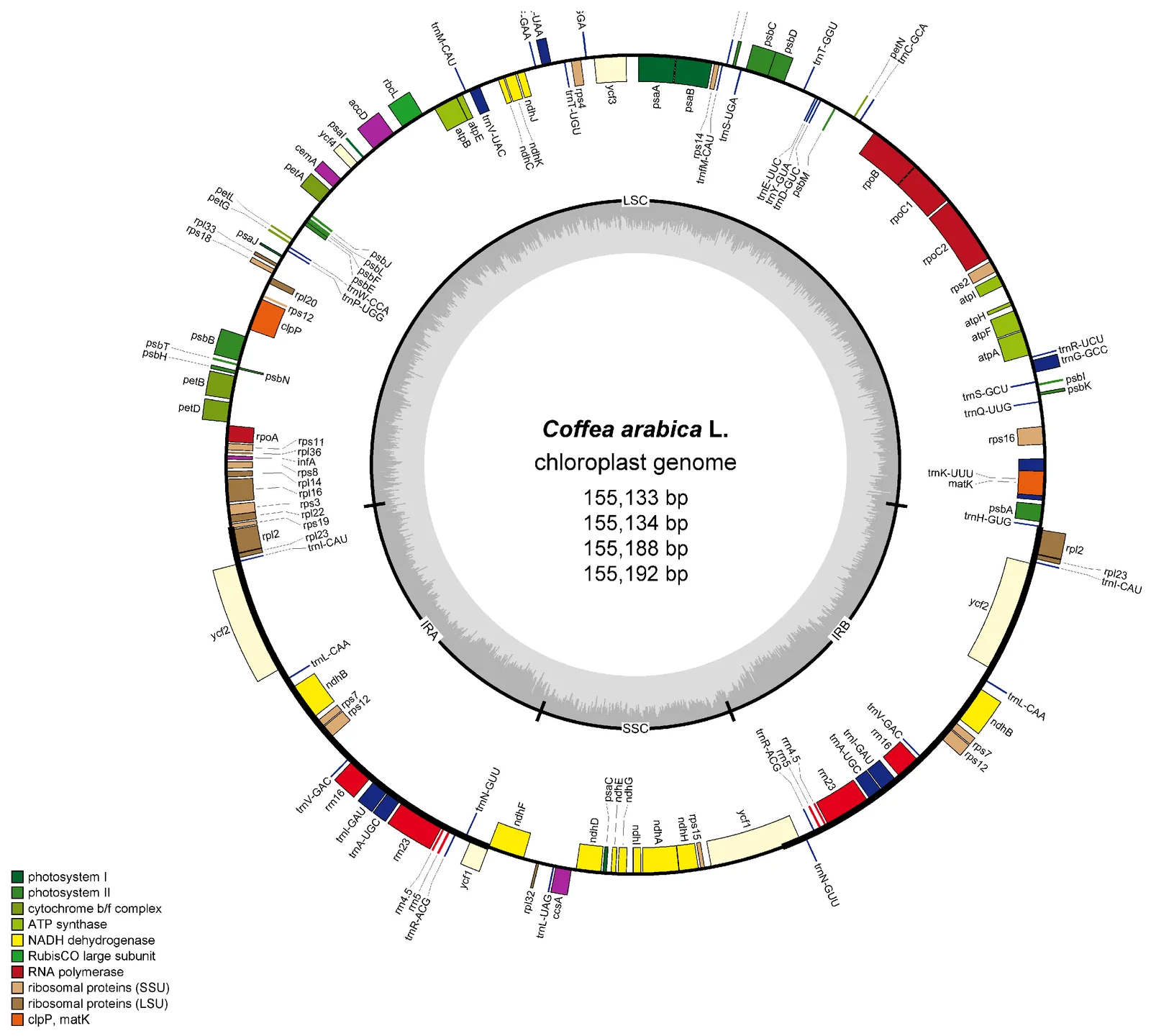
Chloroplast genome map of *C. arabica* (Outer ring: genes colored by functional categories; inner rings show the four regions (LSC, SSC, IRA, IRB); genome size ranges from 155,133 to 155,192 bp).

In this study, 52 *C. arabica* chloroplast genomes were successfully assembled and annotated (Table S5). All assembled genomes exhibited the typical circular quadripartite structure (Figure 1), comprising a large single-copy (LSC) region, a small single-copy (SSC) region, and a pair of inverted repeats (IRA and IRB), which is consistent with previously reported coffee chloroplast genome architectures [23]. Per-accession assembly statistics are provided in Table S5; all assemblies met the completeness criteria defined in Methods.

The total length of the 52 chloroplast genomes ranged from 155,133 bp to 155,192 bp (mean = 155,170.6 bp; median = 155,188 bp). C_19 had the shortest genome (155,133 bp), whereas C_16, C_21, and C_26 had the longest (155,192 bp). The LSC, SSC, and IR regions spanned 85,159~85,168 bp, 18,137~18,138 bp, and 25,914 or 25,946 bp, respectively. GC content ranged from 37.43% to 37.44% (mean = median = 37.43%), with the IR regions showing the highest GC content (43.00%~43.03%) and the SSC region the lowest (31.27%). A total of 131 genes were identified, including 86 protein-coding genes (PCGs), 37 tRNA genes, and 8 rRNA genes, consistent with published findings [24,25,26,27]. The LSC contained 82 genes (61 PCGs and 21 tRNAs), the SSC contained 12 genes (11 PCGs and 1 tRNA), and each IR contained 18 genes (7 PCGs, 4 rRNA genes, and 7 tRNA genes); all IR genes are present in two copies.

In terms of gene structure, multiple genes contained introns. In the LSC region, *trnK-UUU*, *rps16*, *trnG-GCC*, *atpF*, *rpoC1*, *trnL-UAA*, *trnV-UAC*, *petB*, *petD*, and *rpl16* each contained a single intron, whereas *clpP* and *ycf3* each possessed two introns. In the SSC, *ndhA* had one intron. In the IRs, *rpl2*, *ndhB*, *trnI-GAU*, and *trnA-UGC* each had one intron. Boundary-spanning genes included *rps19* (LSC/IRB), *ycf1* (IRB/SSC and SSC/IRA), and the trans-splicing gene *rps12* (LSC, IRB, and IRA) [28]. These boundary-spanning genes may contribute to the 32-bp difference between the two IR length variants (25,914 bp vs. 25,946 bp) separating C2 and C3 [29]. All C3 accessions carried a 25,946-bp IR, whereas all C2 accessions carried a 25,914-bp IR; C_19 (C1) differed from the 155,134-bp C2 accessions by only 1 bp in total length, and its distinct junction configuration is detailed in Table S6. Because C1 is represented by a single accession, this boundary feature should be regarded as a haplotype characteristic rather than a lineage-level diagnostic.

### 3.2. Codon Usage Bias Analysis

**Figure 2:**
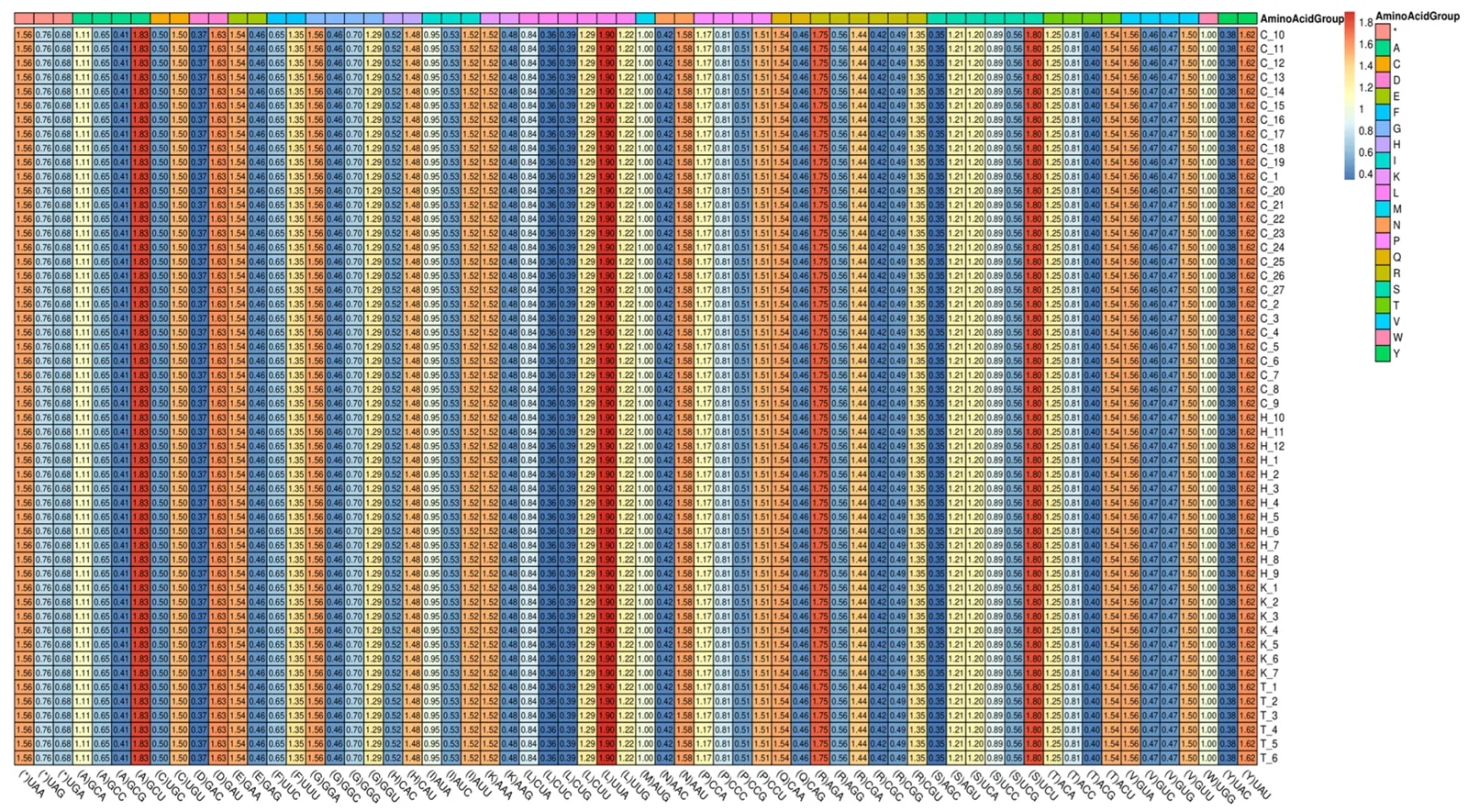
Heatmap of relative synonymous codon usage (RSCU) in 52 chloroplast genomes of *C. arabica.* The color scale ranges from 0.35 to 1.90. Codons with RSCU > 1 tend to end with A/U (e.g., UUA), whereas those with RSCU < 1 tend to end with G/C (e.g., AGC, CUC, GAC). The asterisk (*) denotes the stop codons (UAA, UAG, and UGA). Amino acid groups are labeled with standard single-letter abbreviations.

To investigate the codon usage patterns of the *C. arabica* chloroplast genome, we calculated the relative synonymous codon usage (RSCU) values for all protein-coding sequences (CDSs) across the 52 accessions and generated an RSCU heatmap (Figure 2). The results revealed pronounced codon usage bias in the coffee chloroplast genome [30]. A total of 28 high-frequency codons had RSCU values > 1, most terminating in A or U (e.g., UUA, GUU, GCU, AUU). Conversely, 32 low-frequency codons had RSCU values < 1, predominantly ending in G or C (e.g., AGC, CUC, GAC, UAC) [31]. Among all codons, UUA (leucine) showed the highest RSCU value (1.90), whereas AGC (serine) showed the lowest RSCU value (0.35). For stop codons, UAA, UAG, and UGA yielded RSCU values of 1.56, 0.76, and 0.68, respectively (Figure 2), indicating that UAA is the preferred termination codon. Notably, the 52 *C. arabica* accessions showed remarkable consistency in RSCU values. Minor discrepancies were observed only at the third decimal place for a small subset of codon pairs (e.g., AAA: 1.5160 vs. 1.5156; AAG: 0.4840 vs. 0.4844), corresponding to subtle color variations in the heatmap. This indicates that codon usage bias is highly conserved within this species [32,33]. The prevailing patterns—high-frequency codons preferring A/U endings, low-frequency codons favoring G/C endings, and preferential use of UAA as a stop codon—are common features of terrestrial plant chloroplast genomes. This exceptionally stable codon usage profile is consistent with the AT-rich nucleotide composition of the plastome; because codon-frequency bias alone may substantially reflect nucleotide composition, the pattern is interpreted as a descriptive codon-usage feature rather than demonstrated adaptive optimization of translational efficiency.

### 3.3. Repeat Sequence Analysis

**Figure 3:**
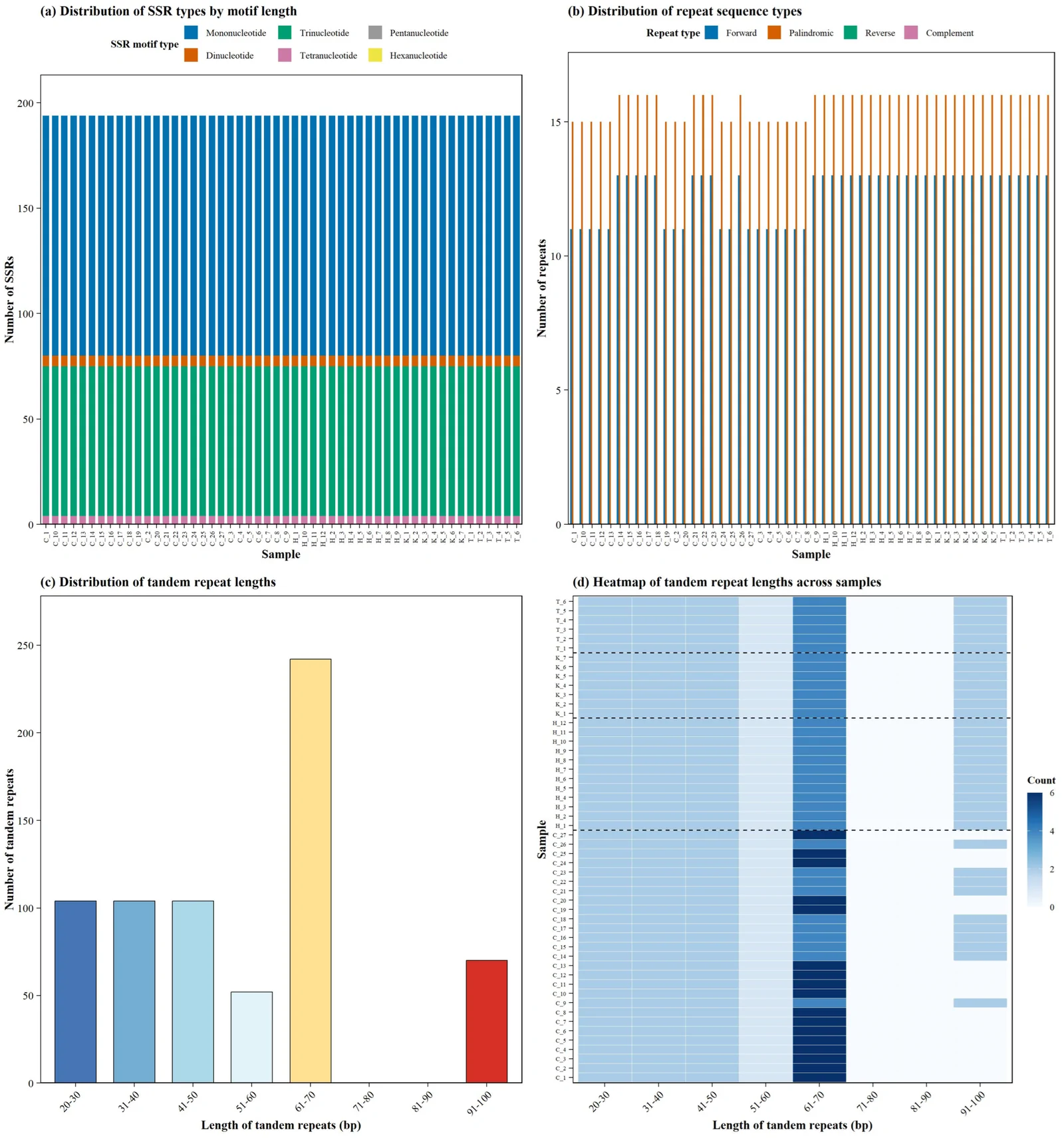
Characterization of repeat sequences in the chloroplast genomes of *C. arabica.* (**a**) Distribution of SSR counts across different motif types (mononucleotide, dinucleotide, and trinucleotide) (stacked bar plot); (**b**) distribution of dispersed repeat types (forward, palindromic, reverse, complement) (grouped bar plot); (**c**) distribution of tandem repeat counts across different length bins (20–100 bp) (bar plot); (**d**) heatmap of tandem repeat length distribution across samples. The color scale indicates repeat abundance, ranging from light blue (0) to dark blue (6). C, H, K, and T represent different coffee cultivar or geographic origin groups. Dashed lines separate the four cultivar groups: C = Geisha, H = Bourbon/Caturra, K = Catimor, and T = Catimor-related accessions.

We systematically characterized simple sequence repeat (SSR) motifs across the 52 *C.* chloroplast genomes, identifying 194 SSR loci per plastome (locus counts by repeat class: 114 mononucleotide, 5 dinucleotide, 71 trinucleotide, 4 tetranucleotide, 0 pentanucleotide, and 0 hexanucleotide loci). The SSR locus count was identical across all 52 accessions. Consistent with most plant chloroplast genomes, mononucleotide repeats predominated numerically (114 loci per plastome), with A/T motifs being the most abundant, averaging 108 per sample. This reflects the strong AT bias characteristic of chloroplast genomes. Among di-, tri-, and tetranucleotide repeats, AAG/CTT and AAT/ATT represented relatively abundant motif classes. Dispersed repeat analysis detected only forward and palindromic repeats (Figure 3b); reverse and complementary repeats were absent, a pattern consistent with the chloroplast genome architecture reported in certain plant species [34]. Palindromic repeats were the most numerous, averaging 15.67 per genome, with repeat lengths concentrated predominantly in the 30–39 bp category. Tandem repeat analysis revealed that the 61–70 bp length class was the most abundant, averaging 4.65 per genome (Figure 3c,d). Notably, tandem repeats of 71–90 bp were not detected. This length distribution profile likely reflects the high conservation and structural stability of the *C. arabica* chloroplast genome [35].

### 3.4. Population Genetic Structure and Lineage Divergence

**Figure 4:**
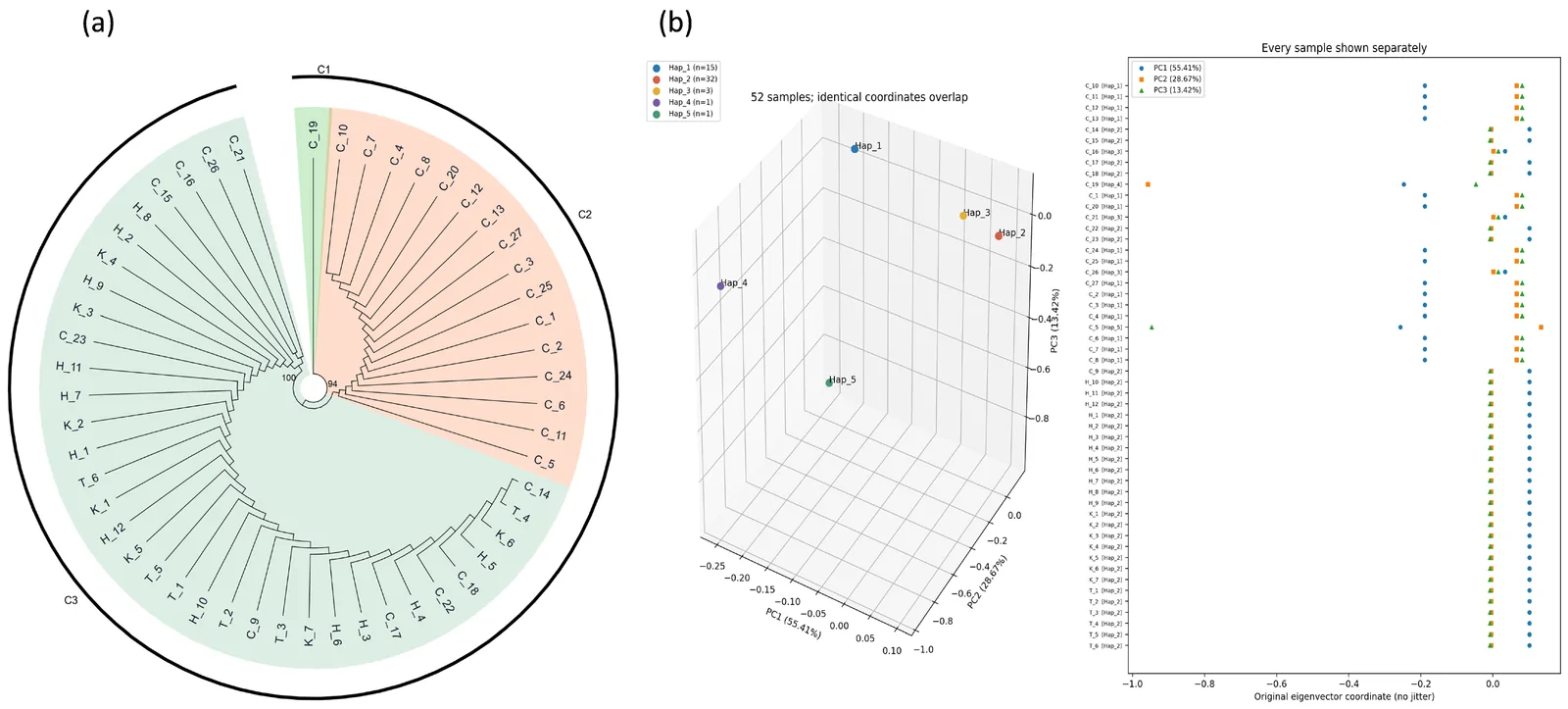
Population genetic structure of *C. arabica* based on complete chloroplast genome sequences: (**a**) unrooted Neighbor-Joining phylogenetic tree with outer ring colors indicating three clusters (C1: green; C2: orange; C3: cyan) and NJ bootstrap/ML ultrafast-bootstrap support values displayed at the major nodes; (**b**) three-dimensional PCA scatter plot of all 52 accessions, with the percentage of variance explained indicated on the PC1, PC2, and PC3 axes.

The unrooted Neighbor-Joining (NJ) tree constructed from complete chloroplast genome sequences (Figure 4a) clearly partitioned the 52 accessions into three genetic clusters: C1 (comprising solely C_19), C2 (encompassing C_1, C_2, C_3, C_4, C_5, C_6, C_7, C_8, C_10, C_11, C_12, C_13, C_20, C_24, C_25, and C_27; 16 accessions in total), and C3 (containing the remaining 35 accessions, including C_9, C_14-C_18, C_21–C_23, C_26, and all H, K, and T series samples). The maximum-likelihood (ML) analysis (best-fit model selected by ModelFinder: F81+F) recovered a topology consistent with the NJ tree; the three clusters were supported by ultrafast bootstrap values of 94% and 100% at the corresponding nodes (the corresponding NJ bootstrap proportions were 80% and 99%; Figure 4a). Because only 15 polymorphic sites were available, branch support was evaluated explicitly and is displayed at all major nodes; relationships within C3 were less strongly supported than the C1/C2/C3 split itself. C1 contained only one accession (C_19, Geisha/G13). The extremely low overall genetic variation observed here is consistent with previous reports for *C. arabica* [36,37].

Principal component analysis (PCA) (Figure 4b) corroborated the NJ clustering pattern. All 52 accessions were plotted individually at the accession level; PC1, PC2, and PC3 explained 55.4149%, 28.6727%, and 13.4195% of the total variance, respectively (Table S10; accession-level coordinates in Table S11). PC1 distinctly separated C1/C2 from C3 and PC2 differentiated C1 from C2, while PC3 primarily captured subtle internal differentiation within C3. Notably, the three samples with the longest genomes (C_16, C_21, and C_26) clustered at the periphery of C3 in the accession-level PCA space (Figure 4b). Independent reanalysis reproduced the same explained variances and PC coordinates (coordinate correlations = 1.0000; Table S12). The three maternal clusters were numerically concordant with the three nuclear genomic groups previously reported for *C. arabica* [38].

### 3.5. Haplotype Network and Maternal Evolutionary History

**Figure 5:**
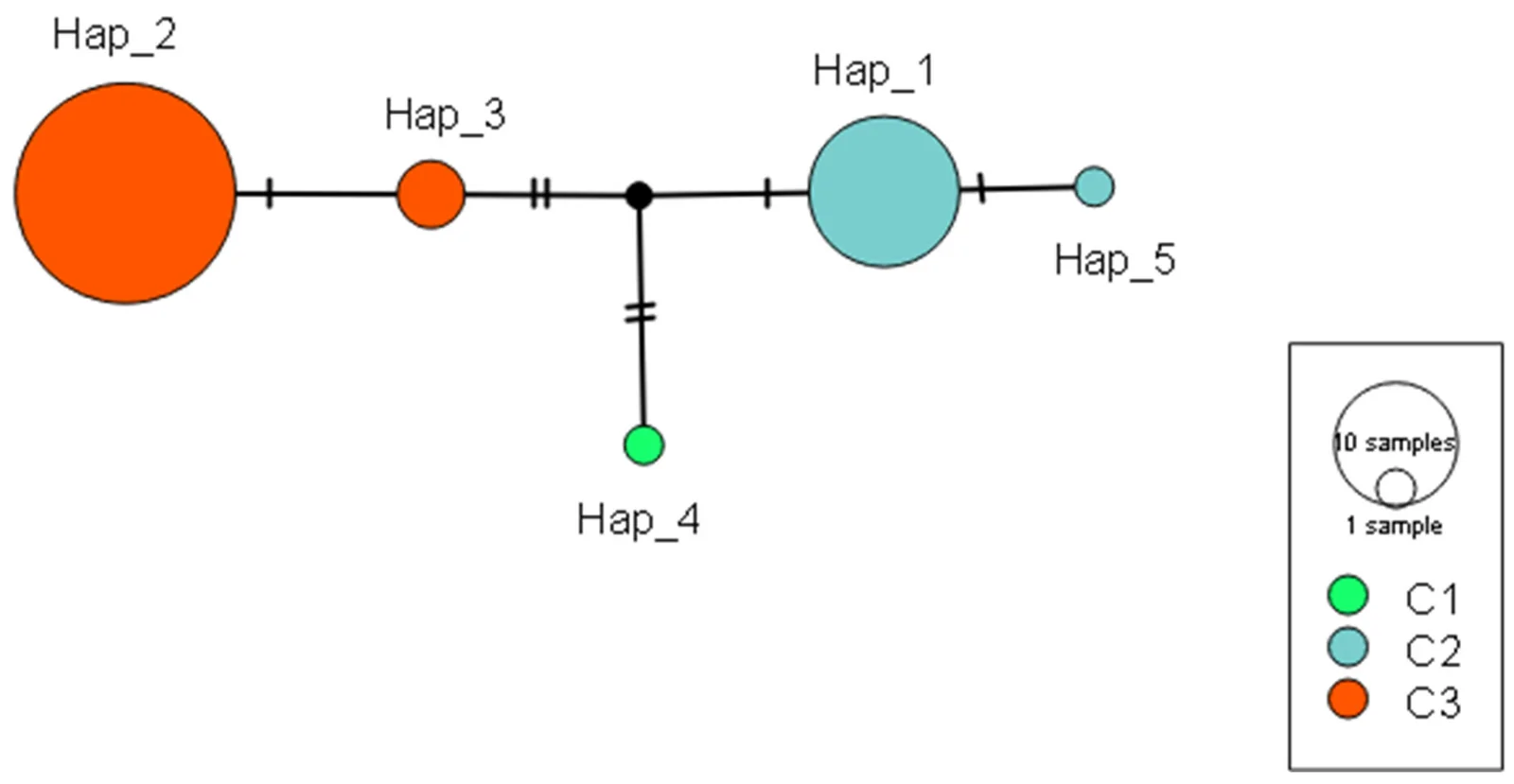
Haplotype network of *C. arabica* based on 15 nucleotide polymorphic sites showing evolutionary relationships among five haplotypes (Hap1–Hap5). Circle size is proportional to haplotype frequency, lines represent mutation steps, colors correspond to genetic clusters (C1: green; C2: orange; C3: cyan).

Haplotype network analysis based on 15 nucleotide polymorphic sites resolved the 52 accessions into five haplotypes (Figure 5). C1 (C_19) corresponded to a distinct haplotype (Hap_4), which showed the greatest genetic distance from all other haplotypes. C2 comprised two haplotypes (Hap_1 and Hap_5) connected by a single mutational step. C3 contained two haplotypes (Hap_2 and Hap_3); Hap_2 was the central haplotype, encompassing the majority of C3 accessions together with all H, K, and T series samples and displaying a star-like pattern, whereas Hap_3 was restricted to a minority of samples. Overall, the network revealed clear mutational connections among haplotypes within C2 and C3 and the distant separation of Hap_4 (C1).

### 3.6. Regional Distribution Characteristics of Chloroplast Genome Variation

**Figure 6:**
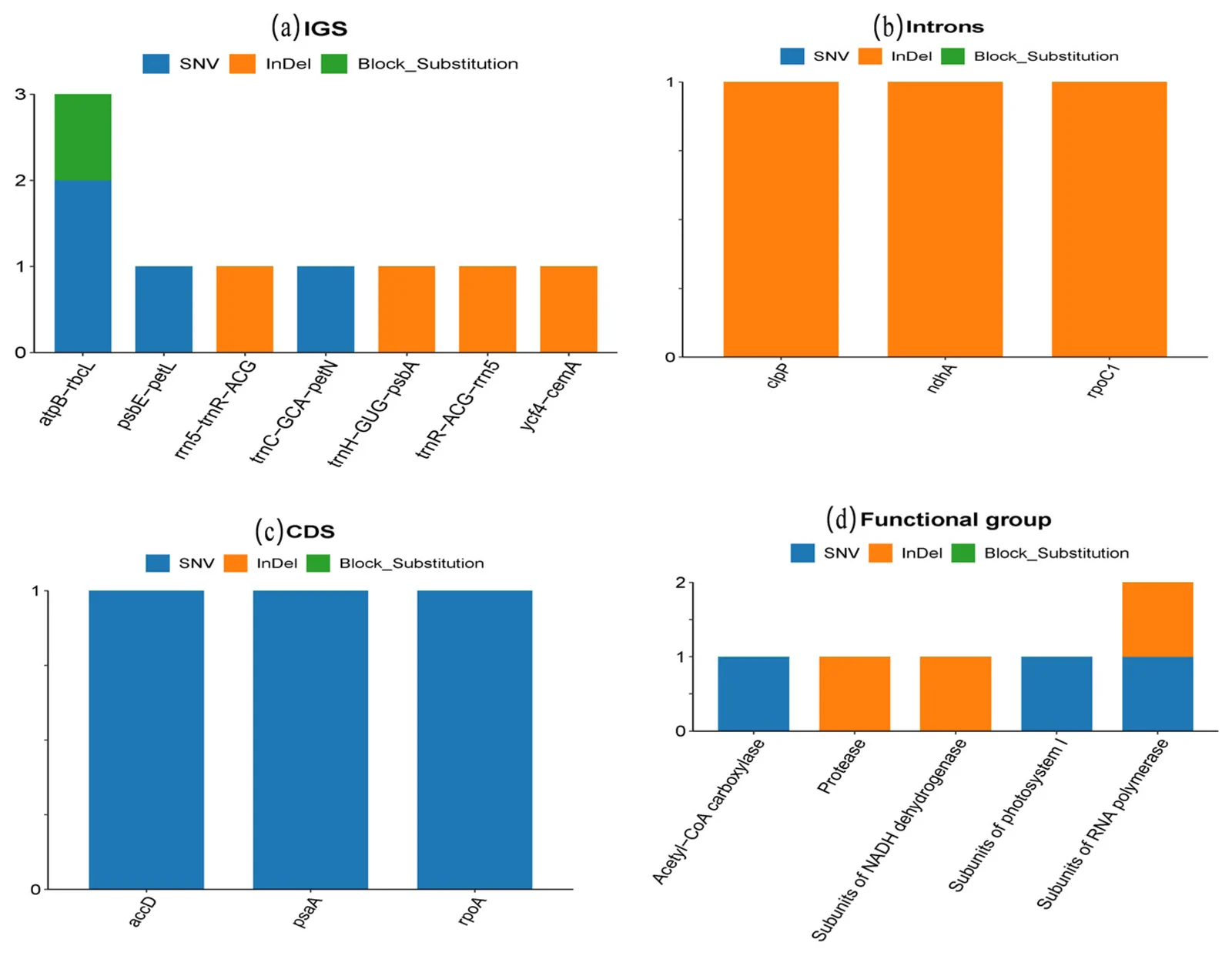
Regional distribution of 15 nucleotide polymorphic sites in the chloroplast genome of *Coffea arabica*: (**a**) intergenic spacers (IGSs); (**b**) introns; (**c**) coding sequences (CDSs); (**d**) functional gene categories. SNV in blue, InDel in orange, Block Substitution in green.

Whole-genome alignment of the 52 chloroplast genomes identified 15 polymorphic records, corresponding to 14 independent variant events after merging V05 and V06 into the block substitution V15 (Figure 6; Table S13). The 15 records comprised 7 single nucleotide variants (SNVs), 7 insertions/deletions (InDels), and 1 block substitution. Regarding genomic regional distribution, intergenic spacer (IGS) regions harbored the richest polymorphism (9 sites, 60.00%), followed by protein-coding regions (CDSs, 3 sites, 20.00%) and intron regions (3 sites, 20.00%). All CDS variants were SNVs (accD, psaA, and rpoA, one each). Annotation of the three CDS SNVs against their reading frames showed the following: the accD SNV (A > G at position 58,777) was nonsynonymous (codon 206: AAA → AGA, Lys → Arg), the psaA SNV (T > G at position 40,810) was synonymous (codon 533: GTA → GTC, Val → Val), and the rpoA SNV (C > T at position 79,101) was nonsynonymous (codon 305: GAT → AAT, Asp → Asn) (Table S4). The scarcity of coding-region variation is consistent with purifying selection, although a formal test of selection (e.g., dN/dS) was not performed. Specifically, all intronic variants were InDels (*clpP*, *ndhA*, and *rpoC1*, one each). IGS regions accumulated the greatest variation, with the atpB-rbcL region being particularly prominent (2 SNVs and 1 block substitution; V05 + V06 merged as V15). Analysis by functional gene category revealed that RNA polymerase subunit genes (*rpoA*, *rpoC1*) exhibited the richest variation, containing one SNV and one InDel. Genes encoding acetyl-CoA carboxylase, protease, NADH dehydrogenase subunits, and photosystem I subunits each contained one SNV or InDel.

## 4. Discussion

This study presents the first systematic analysis of population genetic structure in 52 *C. arabica* accessions based on complete chloroplast genome sequences, identifying three distinct maternal lineages (C1, C2, and C3). The coffee chloroplast genome exhibited remarkable conservation in length (155,133–155,192 bp), GC content (37.43–37.44%), and gene number (131), consistent with reported features of higher plant chloroplast genomes [39,40]. The pronounced A/U codon usage bias is consistent with the AT-rich nucleotide composition of the plastome; given the absence of expression data, this pattern is interpreted as a codon-usage feature that may largely reflect nucleotide composition rather than demonstrated optimization of translational efficiency. C1 is represented by a single accession (C_19, Geisha/G13, collected in Pu’er, Yunnan) and is described as a unique, highly divergent chloroplast haplotype rather than a well-characterized population. The predominance of C3 among the sampled accessions is consistent with the widespread dissemination of the Bourbon/Typica cultivated group [37]; however, all 52 accessions were sampled in Yunnan and cultivar representation was highly uneven (27 of 52 accessions were Geisha), which limits population-level inference.

The three maternal clusters resolved here are numerically concordant with the three genetic groups identified by Salojärvi et al. [37] and with the three groups reported for a Chinese germplasm collection based on RAD-seq [38]. However, because the studies differ in marker inheritance, genomic coverage, sampling strategy, and geographic representation—and because no shared reference accessions were genotyped with nuclear markers in the present study—this correspondence should be treated as a hypothesis for future cytonuclear analysis rather than a demonstrated equivalence. While Salojärvi et al. [37] characterized these groups based on biparentally inherited nuclear SNPs, our chloroplast genome approach provides unambiguous evidence for maternal lineage differentiation, offering a complementary perspective on the evolutionary history of *C. arabica*. The central haplotype structure revealed by network analysis reflects the non-uniform retention of maternal diversity during domestication [36], although the unrooted network cannot establish the direction of haplotype derivation.

C_16, C_21, and C_26 carry chloroplast haplotypes that are genetically close to the C3 cluster but occupy peripheral positions in PCA space. Because the plastome is maternally inherited, these positions reflect their chloroplast haplotypes only. In addition, several of our samples are multiple individual plants of the same clonally maintained cultivar or breeding line and may share identical maternal haplotypes, which should be taken into account when interpreting haplotype frequencies, and both sample-level and unique-accession-level summaries are provided (Tables S2–S4). SSR analysis revealed A/T mononucleotide repeats that represent candidate loci for developing molecular markers, while the predominance of the 15 polymorphic sites in IGS regions (60%) and the elevated polymorphism in atpB-rbcL and trnL-trnF regions render these candidate regions for marker development in Coffea [41,42,43]; the polymorphism and discriminatory power of these candidate SSR and IGS markers remain to be validated experimentally. Among functional gene categories, RNA polymerase genes exhibited relatively more variation than other categories in this small dataset; given the small number of variants and the absence of dN/dS analysis, this pattern should be interpreted descriptively rather than as evidence for relaxed selective constraint [44].

In the context of the recently published chromosome-scale nuclear genome assembly, our study provides a cytoplasmic counterpart that can help dissect cytoplasmic–nuclear coevolution in *C. arabica*. The extremely low genetic variation observed across our 52 chloroplast genomes—only 15 polymorphic sites despite the inclusion of diverse cultivars and breeding lines—is consistent with the severe genetic bottleneck resulting from the single allopolyploidization event at the origin of *C. arabica* approximately 350,000~610,000 years ago [45]. Salojärvi et al. [37] further demonstrated that modern cultivars underwent additional post-domestication bottlenecks, and our maternal lineage analysis is compatible with this scenario: the star-like pattern of Hap_2 within C3 is compatible with, but does not by itself demonstrate, the proliferation of a narrow maternal founder population; uneven sampling and the clonal propagation of cultivars can generate similar topologies. The presence of the C1 and C2 lineages in the sampled Yunnan germplasm, although rare in global collections, suggests that the Chinese coffee genetic resources sampled here retain maternal diversity that may warrant conservation attention; however, given the limited and uneven sampling, expanded surveys are needed before firm conservation priorities can be set.

This study is limited to maternal haplotype information derived from the chloroplast genome and thus cannot elucidate the whole-genome evolutionary history at the nuclear level. Future research should integrate nuclear genome SNPs, transcriptomic data, and phenomic data to construct a pan-genome atlas [37]. The availability of the high-quality *C. arabica* reference genome now facilitates such integrative analysis, enabling the mapping of chloroplast haplotypes onto nuclear genomic backgrounds to test the hypothesized correspondence between the plastid clusters and nuclear groups. In addition, expanding sampling to include wild Ethiopian populations and diploid relatives such as *C. eugenioides* and *C. canephora* [46]—which could also serve as outgroups to root the plastome phylogeny—would help test whether the C1 haplotype is related to the maternal donor of the allopolyploid event, thereby addressing a long-standing question in coffee evolutionary biology. Comparative chloroplast genomics involving these diploid progenitors, as initiated by Charr et al. [47] for *C. canephora*, would complement such analyses.

## 5. Conclusions

In this study, we assembled 52 complete chloroplast genomes of *C. arabica* and resolved three maternal clusters (C1–C3) and five haplotypes. C1 is a unique, highly divergent chloroplast haplotype represented by a single Geisha/G13 accession; C2 and C3 represent differentiated maternal lineages within the cultivated gene pool, with C3 encompassing the majority of accessions. A total of 15 polymorphic records, corresponding to 14 independent variant events, were detected—nine in intergenic spacer (IGS) regions, three in coding regions, and three in introns—together with 194 SSR loci per plastome, providing candidate maternal markers whose utility for germplasm authentication and breeding requires experimental validation. Because chloroplast data capture only maternal history, the correspondence between the plastid clusters and previously reported nuclear genomic groups, as well as questions of domestication history, demographic expansion, and allopolyploid origin, remain to be tested by integrating nuclear genomics, expanding sampling to include wild relatives and suitable outgroups.

## Data Availability

The raw sequencing reads are available in the NCBI SRA under BioProject accession numbers PRJNA1157617 and PRJNA1502542. The 52 assembled chloroplast genomes and annotation files have been submitted to NCBI GenBank under submission ID SUB16536206, and the corresponding GenBank accession numbers are QB169597–QB169648 (listed in Table S1).

## Supplementary Materials

The following supporting information can be downloaded at https://www.mdpi.com/article/10.3390/genes17101304/s1.

## Abbreviations

The following abbreviations are used in this manuscript:

CDS	Coding sequence
IGS	Intergenic spacer
IR	Inverted repeat
LSC	Large single-copy
NJ	Neighbor-joining
PCG	Protein-coding gene
PCA	Principal component analysis
RSCU	Relative synonymous codon usage
SSC	Small single-copy
SNP	Single nucleotide polymorphism
SSR	Simple sequence repeat
